# Anti-Inflammatory and Antinociceptive Studies of Hydroalcoholic Extract from the Leaves of *Phyllanthus brasiliensis* (Aubl.) Poir. and Isolation of 5-*O*-β-d-Glucopyranosyljusticidin B and Six Other Lignans

**DOI:** 10.3390/molecules23040941

**Published:** 2018-04-18

**Authors:** Luziane da C. Borges, Raimundo Negrão-Neto, Sônia Pamplona, Luanna Fernandes, Mayra Barros, Enéas Fontes-Júnior, Cristiane Maia, Consuelo Y. Yoshioka e Silva, Milton Nascimento da Silva

**Affiliations:** 1 Programa de Pós-Graduação em Química, Instituto de Ciências Exatas e Naturais, Universidade Federal do Pará, Rua Augusto Corrêa, 01, Belém 66075-110, Pará, Brazil; luziane_borges22@yahoo.com (L.d.C.B.); negraoneto@yahoo.com.br (R.N.-N.); sgpamplona@yahoo.com.br (S.P.); yumilton@yahoo.com.br (M.N.d.S.); 2 Programa de Pós-Graduação em Ciências Farmacêuticas, Instituto de Ciências da Saúde, Universidade Federal do Pará, Rua Augusto Corrêa, 01, Belém 66075-110, Pará, Brazil; luannafe@hotmail.com (L.F.); mayraarouckbarros@gmail.com (M.B.); efontes@ufpa.br (E.F.-J.); crismaia@ufpa.br (C.M.); 3 Faculdade de Farmácia, Instituto de Ciências da Saúde, Universidade Federal do Pará, Rua Augusto Corrêa, 01, Belém 66075-110, Pará, Brazil

**Keywords:** *Phyllanthus brasiliensis*, 5-*O*-β-d-glucopyranosyljusticidin B, antinociceptive/anti-inflammatory

## Abstract

The aim of this study was to investigate the chemical composition and the antiinflammatory/antinociceptive properties of the hydroalcoholic extract derived from the leaves of *Phyllanthus brasiliensis* (HEPB) in rodents. A new arylnaphthalene lignan glycoside, 5-*O*-β-d-glucopyranosyljusticidin B, together with six known lignans, were isolated from HEPB. 1D and 2D NMR experiments and HRMS were used to elucidate the structure of the new compound. HEPB toxicity and antinociceptive activity were evaluated through acute oral toxicity and formalin models in mice, respectively. The anti-inflammatory effects of HEPB were assessed using carrageenan- and dextran-induced paw edema models in rats. HEPB showed low toxicity. Oral administration of HEPB reduced paw edema induced by carrageenan, but not by dextran. HEPB and its fractions from FR6 to FR10 (FR6-10) inhibited the neurogenic and inflammatory phases of formalin-induced linking, demonstrating its antinociceptive activity. These results indicated that lignans from *Phyllanthus brasiliensis* exerted antinociceptive/anti-inflammatory effects not related to the histaminergic pathway.

## 1. Introduction

The genus *Phyllanthus* is the largest within the Phyllanthaceae family and one of the most diversified among angiosperm genera, with about 1269 species distributed around the world [1].

In Brazil, the genus *Phyllanthus* is known as ‘quebra-pedra’, ‘erva pombinha’ and ‘arrebenta-pedra’. In previous ethnopharmacological reports, leaves, stems, and roots of the genus have been used for the treatment of many diseases, such as urinary and intestinal infections, diabetes and hepatitis B [2]. Some species within the genus *Phyllanthus* have been the subject of scientific studies and are recognized for their antioxidant and anti-inflammatory properties [3,4].

According to phytochemical studies, the genus *Phyllanthus* presents several organic compounds, such as tannins, lignans, alkaloids, triterpens, flavonoids, lactones and steroids [2]. Previous phytochemical studies of *P. brasiliensis* revealed the presence of triterpenes, cyanogenic compounds, the lignan justicidin B, and the glycoside phyllanthoside [5,6].

*P. brasiliensis* is a species that occurs in northern South America (Colombia, Venezuela, Peru, Guyana, French Guiana and Brazil). In Brazil, it is more common in the lowland forests of Acre, Amapá, Amazonas, Pará, and Roraima [1,7,8].

In northwest Guyana, *P. brasiliensis* is used in traditional medicine through the application of macerated leaves. The leaves are heated above a fire and applied as a poultice to the painful bites of the munuri ant (*Paraponera clavata*), which may suggest the topical analgesic activity of the plant [5].

Due to the importance and activities cited for some plants belonging to the genus *Phyllanthus*, as well as the traditional use of leaves from *P. brasiliensis* in Guyana, the present study aims to investigate the anti-inflammatory and antinociceptive activities of *P. brasiliensis*, seeking to validate the use of this plant as a pain relief agent, and furthermore, contribute to the chemical knowledge of this plant through the isolation and characterization of its compounds. We demonstrated here, that the hydroalcoholic extract and a fraction from the leaves of *P. brasiliensis* exerted antiedematogenic, anti-inflammatory, and antinociceptive effects in rodents. In fact, these biological activities have been previously reported for the genus *Phyllanthus*; however, there is a lack of studies for the species used in the present work.

## 2. Results and Discussion

### 2.1. Pharmacological Assays

#### 2.1.1. Carrageenan-Induced Rat Paw Edema

**Figure 1:**
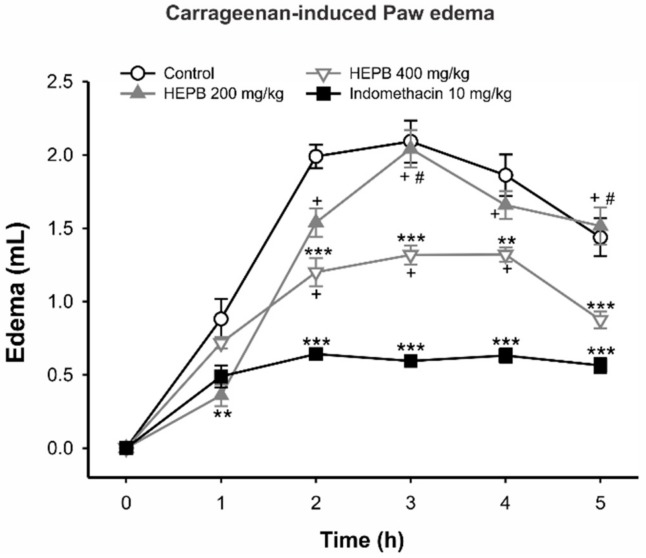
The effects of treatment with *Phyllanthus brasiliensis* (HEPB) on the carrageenan-induced paw edema in rats. Groups: Control (vehicle); HEPB at 200 mg/kg and 400 mg/kg (orally); Indomethacin (IDM) 10 mg/kg (orally). Results expressed as mean ± S.E.M. of edema volume (*n* = 6 per group). ** *p* < 0.01 versus control group; *** *p* < 0.001 versus control group; + *p* < 0.05 versus IDM group; # *p* < 0.05 versus HEPB 400 mg/kg group. Repeated measures one-way ANOVA, followed by Bonferroni post hoc test.

In the antiedematogenic test, Figure 1 shows that a *Phyllanthus brasiliensis* (HEPB) extract of 200 mg/kg reduced the edema volume of the paw 1 h after noxious stimulus (carrageenan). HEPB 400 mg/kg diminished edema for the second hour of the test (*p* < 0.001) and the anti-edematogenic activity was sustained until the end of the test (3 h, *p* < 0.001; 4 h, *p* < 0.01; 5 h, *p* < 0.001), with similar potency to indomethacin in the last hour. As expected, indomethacin started to reduce rat paw edema at the second stage of the test (*p* < 0.001). As mentioned previously, Phyllanthus is a source of naturally occurring therapeutic agents distributed throughout tropical countries, including Brazil [2], and the infusion of their leaves, stems and roots has been used in native medicine to treat several diseases. Although the extracts and compounds of these plants have been investigated in several anti-inflammatory and antinociceptive paradigms, these properties of *P. brasiliensis* have not yet been reported [2,9].

Therefore, our group firstly investigated the antiedematogenic activity of *P. brasiliensis* through the paw edema test induced by carrageenan. The carrageenan paradigm is a suitable experimental animal model for evaluating the anti-edematous effect of biocompounds and widely used to investigate the pathophysiology of the inflammatory response, as well as for characterization of novel anti-inflammatory drugs [10,11]. The Carrageenan triggers an acute inflammatory process in three distinct phases: the first phase (0–90 min) is related to histamine and serotonin release, the second phase involves the release of cytokines, and the third phase (150–360 min) is associated with prostaglandin overproduction [12].

Our results demonstrated HEPB antiedematogenic effects, mainly at a concentration of 400 mg/kg, ratifying its anti-inflammatory activity. These effects were observed after the second hour of the test until the fifth hour, which may suggest that HEPB interferes with cytokine production, possibly by reducing the concentration of proinflammatory mediators, such as bradykinin and prostanoids [12,13]. Nevertheless, further studies are needed to elucidate the exact mechanism of action.

#### 2.1.2. Dextran-Induced Rat Paw Edema

**Figure 2:**
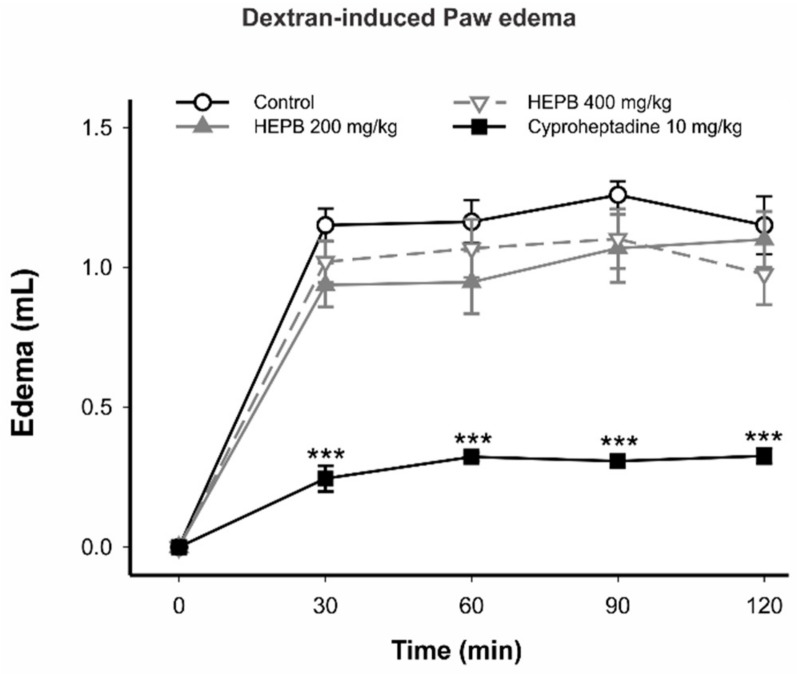
Effects of treatment with HEPB on the dextran-induced paw edema in rats. Groups: control (vehicle); HEPB at 200 mg/kg and 400 mg/kg (orally); Cyproheptadine 10 mg/kg (orally). Results expressed as mean ± S.E.M. of edema volume (*n* = 5 per group). *** *p* < 0.001 versus control group. Repeated measures one-way ANOVA, followed by Bonferroni post hoc test.

Figure 2 shows that 200 mg/kg and 400 mg/kg of HEPB did not present activity in the dextran-induced paw edema model. Cyproheptadine, a first-generation antihistamine drug, reduced rat paw edema at all stages of the test (*p* < 0.001). In any case, to exclude the involvement of histamine and serotonin pathways in the anti-inflammatory activity, we carried out the paw edema test induced by dextran, which has histamine as a proinflammatory mediator [14]. HEPB did not present any antiedematogenic effects in this model. This result excludes an anti-histaminergic-based mechanism in the anti-inflammatory effect of HEPB. In fact, previous studies have highlighted the anti-inflammatory activity of the genus *Phyllanthus* due to the inhibition of the proinflammatory mediators, predominantly prostaglandin [3,15].

#### 2.1.3. Formalin Test

**Figure 3:**
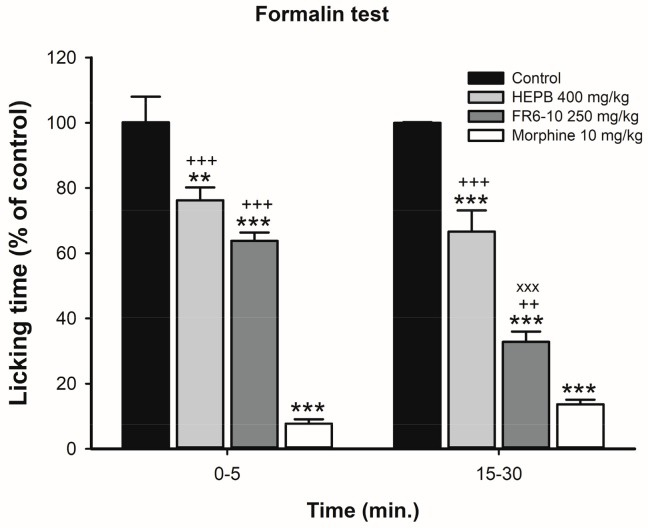
Effects of treatment with HEPB on neurogenic (0–5 min) and inflammatory (15–30 min) phases of the formalin test. Groups: Control (vehicle); HEPB 400 mg/kg (orally); fractions from FR6 to FR10 (FR6-10) 250 mg/kg; and morphine 10 mg/kg (subcutaneously). Results expressed as mean ± S.E.M. of licking time (*n* = 6–8 per group). ** *p* < 0.01 versus control group; *** *p* < 0.001 versus control group; ++ *p* < 0.01 versus Morphine group; +++ *p* < 0.001 versus Morphine group; xxx *p* < 0.001 versus HEPB 400 mg/kg group. One-way ANOVA, followed by Bonferroni post hoc test.

HEPB 400 mg/kg and FR6-10 250 mg/kg reduced the licking time in the first phase (neurogenic pain) and in the second phase (inflammatory pain) in the formalin-induced nociception model (HEPB: *p* < 0.05 and *p* < 0.0001, respectively; FR6-10: *p* < 0.05 and *p* < 0.0001, respectively). Morphine induced significant inhibition in both the first and second phases in the formalin-induced nociception model. These results suggest the antinociceptive activity of the HEPB and the FR6-10 (Figure 3). The ethnopharmacological use of *P. brasiliensis* as a poultice applied to the painful bites of the munuri ant (*Paraponera clavata*) suggests a probable antinociceptive effect. For that reason, we carried out the formalin test using the most effective concentration of HEPB in the anti-inflammatory test (400 mg/kg), as well as the fractions from FR6 to FR10. Our results demonstrated that HEPB and FR6-10 display antinociceptive activity in neurogenic (first phase) and inflammatory (second phase) pain, which provides support for its ethnopharmacological use.

Studies have demonstrated antinociceptive and anti-inflammatory activity related to the genus *Phyllanthus*. Actually, Santos et al. [9] reported the noticeable antinociception effects of *P. amarus*, *P. orbiculatus*, *P. fraternus* and *P. stipulatus* in a dose-dependent manner for the chemical paradigms of nociception, including formalin. The study by Santos et al. reaffirms antinociceptive activity related to the genus, finding that other *Phyllanthus* species have been previously described exerting such activity [16].

Interestingly, not all species of *Phyllanthus* display anti-inflammatory effects. Extracts of *P. stipulatus*, *P. orbiculatus* and *P. simplex* were able to inhibit paw edema, whether or not the latter was associated with the last phase of the formalin test [3,9]. In addition, some species did not present anti-inflammatory nor antinociceptive activities [9]. Nonetheless, *P. brasiliensis* displayed long-lasting anti-inflammatory effects, which persisted for five hours after acute inflammation induced by carrageenan. *P. brasiliensis* also elicited pronounced antinociceptive activity on the neuropathic- and inflammatory-based pain. 

Besides its anti-inflammatory/antinociceptive activities, security is mandatory among bioactive compounds. In this sense, we performed toxicological tests for *P. brasiliensis*.

#### 2.1.4. Acute Oral Toxicity

In the safety test, the dose of 2000 mg/kg was administered to five animals. No deaths were observed. In this sense, another five animals received 5000 mg/kg of HEPB and were observed for 14 days. Similarly, no weight loss or death was detected.

During the hippocratic screening (animal behavior/depressive alterations), parameters relating to aggression, apathy, ataxia, cyanosis, piloerection, tremors, and convulsions were not observed. Other parameters such as diarrhea, hematuria, sedation, and respiratory discomfort were also not noted. From these results, the safety of HEPB when administered orally can be evidenced. This is also the first time that oral toxicity in vivo was evaluated for this species. Thus, we can affirm that the leaf extract (HEPB) lacked a toxic oral effect. 

### 2.2. General Chemistry

**Figure 4:**
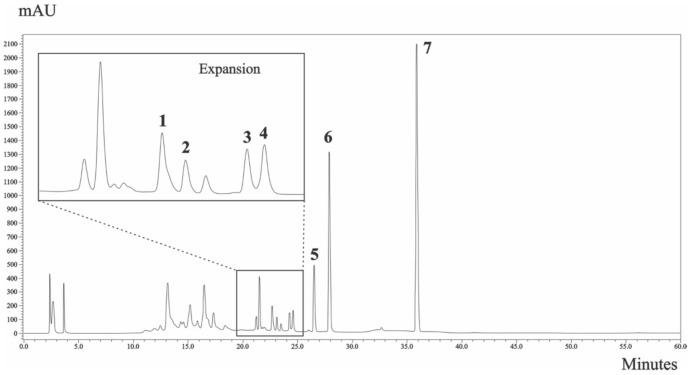
HPLC chromatogram of HEPB at 258 nm. Arabelline (**1**) (peak 1), 4-*O*-β-d-apiofuranosyl-(1′′′→6″)-β-d-glucopyranosyldiphyllin (**2**) (peak 2), 5-*O*-β-d-glucopyranosyljusticidin B (**3**) (peak 3), cleistanthin B (**4**) (peak 4), phyllanthostatin A (**5**) (peak 5), tuberculatin (**6**) (peak 6) and justicidin B (**7**) (peak 7). Chromatographic conditions are described in the Methods section.

The HPLC chromatogram profile for HEPB is shown in Figure 4. 

**Figure 5:**
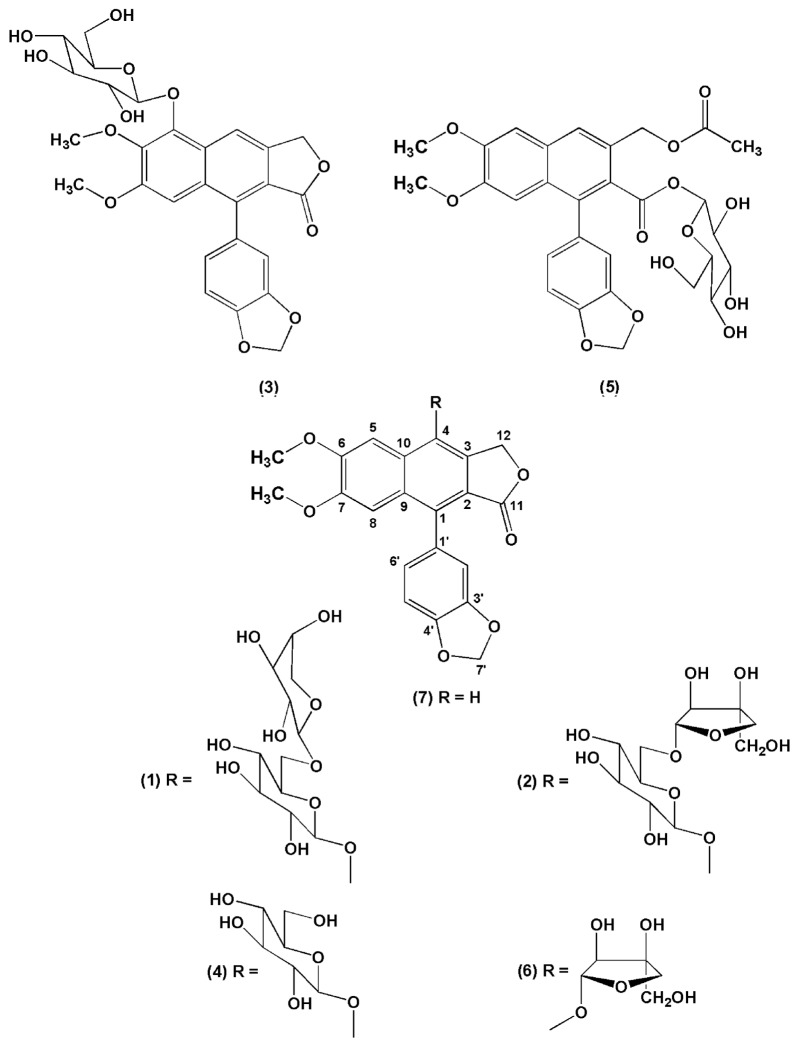
Structures of compounds **1**–**7** isolated from HEPB.

The structures of compounds isolated from HEPB (Figure 5) were established based on spectroscopic data (1D and 2D NMR), mass spectrometry and direct comparison with previously published data.

### 2.3. Isolation of Compounds

The present study investigated the hydroalcoholic extract from the leaves of the *P. brasiliensis*, from which seven arylnaphtalene lignans were isolated. A new compound 5-*O*-β-d-glucopyranosyljusticidin B (**3**) together with six known lignans: arabelline (**1**), 4-*O*-β-d-apiofuranosyl-(1′′′→6″)-β-d-glucopyranosyldiphyllin (**2**), cleistanthin B (**4**), phyllanthostatin A (**5**), tuberculatin (**6**) and Justicidin B (**7**).

Compound **3**, a new substance isolated here, was obtained as a white amorphous solid. Its molecular formula C_27_H_26_O_12_ was established by the observation of the [M + H]^+^ ion at *m*/*z* 543.1503 in the positive ESI-TOF-MS. The IR spectrum indicated the existence of hydroxyl (3370 cm^−1^), γ-lactone (1774 cm^−1^) and aromatic (1619 cm^−1^) bands [17]. The ^1^H-NMR spectrum of compound **3** (Table 1) displayed the presence of three signals at δ 6.78 (d, *J* = 1.5 Hz, H-2′), 6.95 (d, *J* = 7.8 Hz, H-5′) and 6.75 (dd, *J* = 7.8, 1.5 Hz, H-6′), typical of ABX system protons and two singlets, one for each proton, at δ 6.98 and 8.45 in the aromatic region. In the aliphatic region, a singlet was observed at δ 5.43, which was attributed to a γ-lactone methylene group; two signals at δ 6.03 and 6.04 coupling with each other (*J* = 1.2 Hz) assigned to a deoxygenated methylene group and two singlets at δ 3.74 and 3.97, characteristic of methoxyl groups (for more details see Supplementary Materials).

**Figure 6:**
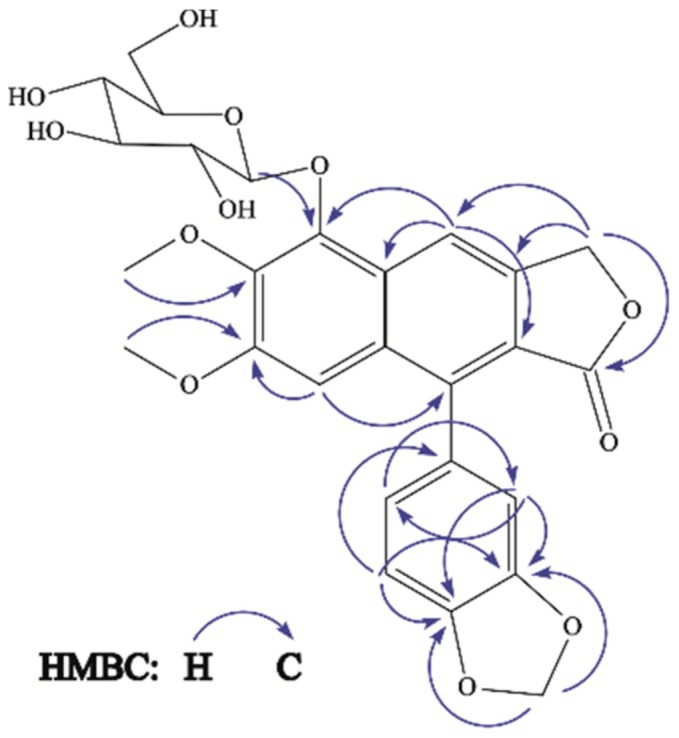
Key HMBC correlations of compound **3**.

The ^13^C-NMR spectrum showed the presence of 26 carbon atoms (two equivalent carbons). The presence of two methoxyl groups displayed by the ^1^H-NMR spectrum at δ 3.74 and 3.97 was confirmed by its ^13^C resonances at δ 56.1 and 62.0. The signal at δ 62.0 suggests that one of the methoxyl groups is diortho-substituted. The presence of a γ-lactone methylene group was confirmed by a ^13^C-chemical shift of a carbonyl carbon at δ 172.3 and a methylene carbon at δ 70.1. The ^1^H-NMR spectrum also exhibited a series of signals between δ 3.20–5.16 characteristic of a sugar moiety. The presence of one anomeric proton at δ 5.16 (d, *J* = 7.8 Hz) suggested the presence of a sugar unit, which was assigned by means HSQC, HMBC and ^1^H-^1^H COSY correlations. The ^13^C-NMR signals at δ 105.7, 75.7, 77.9, 71.3, 78.3 and 62.2 are characteristic of a glucopyranosyl group [18]. The anomeric proton showed HMBC correlation to C-5 (δ 145.4), suggesting that the sugar part has linked to the aglycone part at the C-5 position (Figure 6). The large coupling constant at *J* = 7.8 Hz indicated the β anomeric configuration in the glucopyranosyl moiety. The above discussed spectral evidence led to the identification of a new compound characterized as 5-*O*-β-d-glucopyranosyljusticidin B (**3**). Compounds **1**, **2**, **4**, **5**, **6** and **7** were identified as arabelline [19], 4-*O*-β-d-apiofuranosyl-(1′′′→6″)-β-d-glucopyranosyldiphyllin [17], cleistanthin B [19], phyllanthostatin A [20], tuberculatin [21] and justicidin B [22], respectively by comparison of their spectral data with the literature values.

Compound **1**. The molecular formula was determined as C_32_H_34_O_16_, based on positive-ion HR-TOF-MS (*m*/*z* 697.1745 ([M + Na]^+^; calcd. 697.1745)). ^1^H-NMR (C_5_D_5_N, 300 MHz) δ (ppm): 8.85 (1H, s, H-5), 7.32 (1H, s, H-8), 5.76 (1H, d, *J* = 15.3 Hz, H-12a), 6.29 (1H, d, *J* = 15.3 Hz, H-12b), 4.16 (3H, s, OCH_3_-6), 3.67 (3H, s, OCH_3_-7), 7.19 (1H, d, *J* = 1.5 Hz, H-2′), 7.13 (1H, d, *J* = 7.8 Hz, H-5′), 7.04 (1H, dd, *J* = 1.5, 7.8 Hz, H-6′), 5.94 (1H, d, *J* = 1.2 Hz, H-7′a), 6.04 (1H, d, *J* = 1.2 Hz, H-7′b), 5.40 (1H, d, *J* = 7.8 Hz, H-1″), 4.42 (1H, m, H-2″), 4.11 (1H, m, H-3″), 4.26 (1H, m, H-4″), 4.31 (1H, m, H-5″), 4.78 (2H, m, H-6″), 4.81 (1H, d, *J* = 6.6 Hz, H-1′′′), 4.43 (1H, m, H-2′′′), 4.13 (1H, m, H-3′′′), 4.29 (1H, m, H-4′′′), 3.72 (2H, m, H-5′′′). ^13^C-NMR (C_5_D_5_N, 75 MHz) δ (ppm): 136.1 (C-1), 120.0 (C-2), 132.0 (C-3), 146.0 (C-4), 102.7 (C-5), 152.6 (C-6), 151.0 (C-7), 106.5 (C-8), 128.3 (C-9), 130.9 (C-10), 170.4 (C-11), 68.4 (C-12), 56.4 (OCH_3_-6), 55.5 (OCH_3_-7), 129.5 (C-1′), 111.7 (C-2′), 147.9 (C-3′), 148.0 (C-4′), 108.5 (C-5′), 124.5 (C-6′), 101.7 (C-7′), 106.9 (C-1″), 75.2 (C-2″), 77.0 (C-3″), 71.2 (C-4″), 78.2 (C-5″), 69.5 (C-6″), 105.2 (C-1′′′), 72.1 (C-2′′′), 74.3 (C-3′′′), 69.2 (C-4′′′), 66.7 (C-5′′′). Compared with reported data [19], compound **1** was identified as Arabelline.

Compound **2**. The molecular formula was determined as C_32_H_34_O_16_, based on positive-ion HR-TOF-MS (*m*/*z* 697.1735 ([M + Na]^+^; calcd. 697.1745)). ^1^H-NMR (C_5_D_5_N, 300 MHz) δ (ppm): 8.88 (1H, s, H-5), 7.37 (1H, s, H-8), 5.83 (1H, d, *J* = 15.6 Hz, H-12a), 6.37 (1H, d, *J* = 15.6 Hz, H-12b), 4.15 (3H, s, OCH_3_-6), 3.69 (3H, s, OCH_3_-7), 7.24 (1H, d, *J* = 1.8 Hz, H-2′), 7.12 (1H, d, *J* = 8.1 Hz, H-5′), 7.17 (1H, dd, *J* = 1.8, 8.1 Hz, H-6′), 5.94 (1H, d, *J* = 1.2 Hz, H-7′a), 6.04 (1H, d, *J* = 1.2 Hz, H-7′b), 5.42 (1H, d, *J* = 7.8 Hz, H-1″), 4.46 (1H, t, *J* = 8.5 Hz, H-2″), 4.15 (1H, m, H-3″), 4.17 (1H, m, H-4″), 4.30 (1H, m, H-5″), 4.70 (1H, dd, *J* = 2.1, 10.5 Hz, H-6″a), 4.21 (1H, m, H-6″b), 5.71 (1H, d, *J* = 2.4 Hz, H-1′′′), 4.74 (1H, m, H-2′′′), 4.35 (1H, d, *J* = 9.5 Hz, H-4′′′a), 4.57 (1H, d, *J* = 9.5 Hz, H-4′′′b), 4.13 (2H, m, H-5′′′). ^13^C-NMR (C_5_D_5_N, 75 MHz) δ (ppm): 136.3 (C-1), 120.3 (C-2), 131.9 (C-3), 146.3 (C-4), 102.9 (C-5), 152.8 (C-6), 151.2 (C-7), 106.7 (C-8), 128.5 (C-9), 131.2 (C-10), 170.5 (C-11), 68.6 (C-12), 56.4 (OCH_3_-6), 55.6 (OCH_3_-7), 129.8 (C-1′), 111.7 (C-2′), 148.1 (C-3′), 148.2 (C-4′), 108.6 (C-5′), 124.5 (C-6′), 101.9 (C-7′), 107.1 (C-1″), 75.4 (C-2″), 77.3 (C-3″), 71.8 (C-4″), 78.7 (C-5″), 69.2 (C-6″), 111.1 (C-1′′′), 78.0 (C-2′′′), 80.6 (C-3′′′), 75.3 (C-4′′′), 65.7 (C-5′′′). Compared with reported data [17], compound **2** was identified as 4-*O*-β-d-apiofuranosyl-(1′′′→6″)-β-d-glucopyranosyldiphyllin.

Compound **4**. The molecular formula was determined as C_27_H_26_O_12_, based on positive-ion HR-TOF-MS (*m*/*z* 543.1498 ([M + H]^+^; calcd. 543.1503)). ^1^H-NMR (CD_3_OD, 300 MHz) δ (ppm): 8.12 (1H, s, H-5), 7.01 (1H, s, H-8), 5.45 (1H, d, *J* = 15.6 Hz, H-12a), 5.75 (1H, d, *J* = 15.6 Hz, H-12b), 4.00 (3H, s, OCH3-6), 3.70 (3H, s, OCH_3_-7), 6.73 (1H, d, *J* = 1.5 Hz, H-2′), 6.93 (1H, d, *J* = 7.8 Hz, H-5′), 6.72 (1H, dd, *J* = 1.5, 7.8 Hz, H-6′), 6.02 (1H, d, *J* = 1.0 Hz, H-7′a), 6.03 (1H, d, *J* = 1.0 Hz, H-7′b), 4.82 (1H, d, *J* = 7.9 Hz, H-1″), 3.65 (1H, t, *J* = 9.0 Hz H-2″), 3.46 (1H, m, H-3″), 3.41 (1H, m, H-4″), 3.27 (1H, m, H-5″), 3.89 (2H, d, *J* = 11.4 Hz, H-6″). ^13^C-NMR (CD_3_OD, 75 MHz) δ (ppm): 137.4 (C-1), 120.1 (C-2), 132.2 (C-3), 146.3 (C-4), 102.7 (C-5), 153.3 (C-6), 151.6 (C-7), 106.9 (C-8), 128.8 (C-9), 131.8 (C-10), 172.3 (C-11), 69.4 (C-12), 56.7 (OCH_3_-6), 56.0 (OCH_3_-7), 130.0 (C-1′), 111.7 (C-2′), 148.9 (C-3′), 149.0 (C-4′), 108.9 (C-5′), 124.7 (C-6′), 102.5 (C-7′), 106.7 (C-1″), 75.4 (C-2″), 78.1 (C-3″), 71.4 (C-4″), 78.3 (C-5″), 62.6 (C-6″). Compared with reported data [19], compound **4** was identified as Cleistanthin B.

Compound **5**. The molecular formula was determined as C_29_H_30_O_13_, based on positive-ion HR-TOF-MS (*m*/*z* 609.1586 ([M + Na]^+^; calcd. 609.1584)). ^1^H-NMR (CD_3_OD, 400 MHz) δ (ppm): 7.78 (1H, s, H-4), 7.29 (1H, s, H-5), 6.93 (1H, s, H-8), 5.24 (1H, d, *J* = 12.3 Hz, H-12a), 5.39 (1H, d, *J* = 12.3 Hz, H-12b), 2.09 (3H, s, H-14), 3.96 (3H, s, OCH_3_-6), 3.70 (3H, s, OCH_3_-7), 6.87 (1H, m, H-2′), 6.90 (1H, m, H-5′), 6.83 (1H, m, H-6′), 6.03 (2H, s, H-7′), 5.45 (1H, d, *J* = 8.0 Hz, H-1″), 3.27 (1H, m, H-2″), 3.41 (1H, m, H-3″), 3.34 (1H, m, H-4″), 3.35 (1H, m, H-5″), 3.83 (2H, d, *J* = 12 Hz, H-6″). ^13^C-NMR (CD_3_OD, 100 MHz) δ (ppm): 137.3 (C-1), 128.5 (C-2), 127.4 (C-3), 126.7 (C-4), 106.4 (C-5), 150.7 (C-6), 150.4 (C-7), 105.1 (C-8), 127.9 (C-9), 129.9 (C-10), 167.8 (C-11), 64.9 (C-12), 171.6 (C-13), 19.6 (C-14), 54.9 (OCH_3_-6), 54.6 (OCH_3_-7), 131.2 (C-1′), 110.4 (C-2′), 147.4 (C-3′), 147.7 (C-4′), 108.0 (C-5′), 123.7 (C-6′), 101.1 (C-7′), 94.8 (C-1″), 72.4 (C-2″), 76.5 (C-3″), 69.7 (C-4″), 77.2 (C-5″), 61.2 (C-6″). Compared with reported data [20], compound **5** was identified as Phyllanthostatin A.

Compound **6**. The molecular formula was determined as C_26_H_24_O_11_, based on positive-ion HR-TOF-MS (*m*/*z* 513.1392 ([M + H]^+^; calcd. 513.1397)). ^1^H-NMR (CD_3_OD, 300 MHz) δ (ppm): 7.65 (1H, s, H-5), 7.02 (1H, s, H-8), 5.54 (1H, d, *J* = 15 Hz, H-12a), 5.46 (1H, d, *J* = 15 Hz, H-12b), 3.99 (3H, s, OCH_3_-6), 3.71 (3H, s, OCH_3_-7), 6.76 (1H, d, *J* = 1.5 Hz, H-2′), 6.93 (1H, d, *J* = 7.8 Hz, H-5′), 6.74 (1H, dd, *J* = 1.5, 7.8 Hz, H-6′), 6.02 (1H, d, *J* = 1.2 Hz, H-7′a), 6,04 (1H, d, *J* = 1.2 Hz, H-7′b), 5.49 (1H, d, *J* = 3.3 Hz, H-1″), 4.51 (1H, d, *J* = 3.9 Hz, H-2″), 4.33 (1H, d, *J* = 9.7 Hz, H-4″a), 3.92 (1H, d, *J* = 9.7 Hz, H-4″b), 3.67 (2H, s, H-5″). ^13^C-NMR (CD_3_OD, 75 MHz) δ (ppm): 136.8 (C-1), 119.9 (C-2), 130.1 (C-3), 146.2 (C-4), 101.9 (C-5), 153.2 (C-6), 151.7 (C-7), 107.0 (C-8), 131.7 (C-9), 128.3 (C-10), 172.1 (C-11), 68.7 (C-12), 56.5 (OCH_3_-6), 56.0 (OCH_3_-7), 129.9 (C-1′), 111.8 (C-2′), 148.9 (C-3′), 148.9 (C-4′), 108.9 (C-5′), 124.8 (C-6′), 102.6 (C-7′), 112.8 (C-1″), 78.6 (C-2″), 80.3 (C-3″), 75.9 (C-4″), 64.1 (C-5″). Compared with reported data [21], compound **6** was identified as Tuberculatin.

Compound **7**. The molecular formula was determined as C_21_H_16_O_6_, based on positive-ion HR-TOF-MS (*m*/*z* 365.1022 ([M + H]^+^; calcd. 365.1025)). ^1^H-NMR (CDCl_3_, 300 MHz,) δ (ppm): 7.59 (1H, s, H-4), 7.07 (1H, s, H-5), 6.92 (1H, s, H-8), 5.24 (2H, s, H-12), 3.63 (3H, s, OCH_3_-6), 3.88 (3H, s, OCH_3_-7), 6.66 (1H, dd, *J* = 0.6, 1.8 Hz, H-2′), 6.80 (1H, dd, *J* = 0.6, 7.8 Hz, H-5′), 6.64 (1H, dd, *J* = 1.8, 7.8 Hz, H-6′), 5,87 (1H, d, *J* = 1.4 Hz, H-7′a), 5,92 (1H, d, *J* = 1.4 Hz, H-7′b). ^13^C-NMR (CDCl_3_, 75 MHz,) δ (ppm): 139.2 (C-1), 117.7 (C-2), 139.3 (C-3), 118.1 (C-4), 105.8 (C-5), 149.6 (C-6), 151.4 (C-7), 105.3 (C-8), 128.4 (C-9), 133.0 (C-10), 170.6 (C-11), 68.1 (C-12), 55.3 (OCH_3_-6), 55.6 (OCH_3_-7), 128.0 (C-1′), 110.1 (C-2′), 147.2 (C-3′), 147.2 (C-4′), 107.7 (C-5′), 123.0 (C-6′), 100.9 (C-7′). Compared with reported data [22], compound **7** was identified as Justicidin B.

Arabelline (**1**) and phyllanthostatin A (**5**) have been reported by their cytotoxic activity [20,21]. Cleistanthin B (**4**) has shown antihypertensive effects through alpha-adrenergic receptor blockade [23] and/or potent inhibition of Angiotensin I-Converting Enzyme [24]; diuretic effects [25]; and a potential anticancer property [26]. Biological effects of 4-*O*-β-d-apiofuranosyl-(1′′′→6″)-β-d-glucopyranosyldiphyllin (**2**) have not been investigated so far. Tuberculatin (**6**) and justicidin B (**7**) have been reported as producing cytotoxic effects against cancer cell lines [27,28]. However, their potential anti-inflammatory activity has been poorly investigated. 

As a matter of fact, Prieto et al. [29] and Rao et al. [4] have reported tuberculatin (**6**) and justicidin B (**7**) as anti-inflammatory agents in acute inflammation. In the Rao et al. [4] study, spectrophotometric and enzyme-linked immunosorbent assay (ELISA) procedures were used to elucidate the underlying mechanisms of the anti-inflammatory response in culture cells by justicidin B (**7**). It was shown that this compound decreased the production of nitric oxide (NO) due to a reduction in inducible nitric oxide synthase (iNOS) and inhibition of tumor necrosis factor (TNF-α) and interleukin (IL)-12 production. NO, TNF-α and IL-12 play a key role in the acute and chronic inflammatory processes. Excessive production of NO plays a pathogenic role in both acute and chronic inflammations [30], and TNF-α and IL-12 have been reported as the main pro-inflammatory cytokines released during the early phases of the inflammatory processes [31]. Another pro-inflammatory mediator reduced by justicidin B is the nuclear factor kappa B (NF-kB) (Momekov et al.) [32], which plays a pivotal role in the overexpression of proinflammatory genes encoding cytokine and chemokine production (for review see Lawrence) [33]. Such modulatory effects were similar to those elicited by other biocompounds, such as flavonoids (Menghini et al.) [34]. The above findings support the evidence that anti-inflammatory activity is indeed observed by HEPB. Additionally, justicidin E has been pointed out as inhibiting 5-lipoxygenase activity (5-LOX) [35], which is responsible for catalyzing the biosynthesis of leukotrienes (LTs), which consist of the lipid mediators of inflammation and are derived from arachidonic acid [36].

Although tuberculatin (**6**) has been reported as anti-inflammatory, the mechanism of action that underlies its effect has not been clearly described. All of these findings, in addition to our results in vivo, serve as a basis to explain the anti-inflammatory effect of HEPB, mainly through the action of tuberculatin (**6**) and justicidin B (**7**), which were isolated from our *P. brasiliensis* extract.

In conclusion, our results offer scientific evidence to support the ethnopharmacological use of *P. brasiliensis* as an anti-inflammatory and analgesic medicine. Additionally, this study reveals a new compound, 5-*O*-β-d-glucopyranosyljusticidin B.

## 3. Materials and Methods

### 3.1. Pharmacological Assays

#### 3.1.1. Drugs and Chemical Compounds

Cyproheptadine (Sigma-Aldrich, Saint Louis, MO, USA), Acetic acid (Cromato, Diadema, Brazil); Formaldehyde (Dinamica, Diadema, Brazil); Croton oil (Sigma-Aldrich, Saint Louis, MO, USA); Lambda-carrageenan type IV (Sigma-Aldrich, Saint Louis, MO, USA); Morphine sulphate (Cristália, Itapira, Brazil); Indomethacin (Sigma-Aldrich, Saint Louis, MO, USA); Ethanol (Tedia, Fairfield, OH, USA); hexane (Tedia, Fairfield, OH, USA); ethyl acetate (Tedia, Fairfield, OH, USA); acetonitrile (Tedia, Fairfield, OH, USA); and methanol (Tedia, Fairfield, OH, USA).

#### 3.1.2. Animals

Female (*n* = 10) and male (*n* = 48) Wistar rats, two months old and weighing 180–200 g, and male Swiss mice (*n* = 30) weighing 25–35 g, obtained from the Animal Facility, Biological Sciences Institute, Federal University of Pará (UFPA), were used in the biological assays, approved by ethics committee, license number: CEPAN-IEC 562009. They were kept under standard conditions of temperature, humidity, and a light/dark cycle of 12 h (lights on at 7:00 a.m.) with water and food ad libitum. Fluorescent lights (12 lux) were used in the rooms where the experiments were performed.

The research project was approved by the Research Ethics Committee of the Evandro Chagas Institute (CEPAN-IEC) under number 56/2009, which included the mortality aspects of the protocol, and the study was conducted in accordance with the standards of the *Guide for the Care and Use of Laboratory Animals* of the National Institute of Health. 

At the end of each experiment, animals were deeply anaesthetized with a solution of ketamine hydrochloride (90 mg/kg, i.p.) and xylazine hydrochloride (10 mg/kg, i.p.) and euthanized by cervical dislocation. In the present study, death, sickness, or any other symptoms indicative of the euthanasia procedure were not observed before the experimental endpoint. 

HEPB or FR6-10 was dissolved in a 10% ethanol solution and orally administered through an orogastric tube (gavage). The control groups received vehicle (10% ethanol solution) by gavage. All the HEPB or FR6-10 administrations were made an hour before the experimental assays.

#### 3.1.3. Carrageenan-Induced Rat Paw Edema

Acute inflammation was produced in the male *Wistar* rats (*n* = 6 rats per group) as previously described [12] with slight modifications. Briefly, animals were fasted for 2 h and treated with vehicle, HEPB (200 mg/kg and 400 mg/kg) or indomethacin (10 mg/kg, by gavage). One hour later, animals received intraplantar injections of 100 µL of carrageenan (1%, *w*/*v*) in the right hind paw and the same volume of saline (0.9%) in the contra lateral paw. Edema was measured with a plethysmometer (Model 7140, Ugo Basile, Monvalle, Italy) at times 0 (immediately after), 1, 2, 3, 4 and 5 h after intraplantar injection. Results are presented as the change in paw volume (mL) of the right paw in relation to the left paw volume.

#### 3.1.4. Dextran-Induced Rat Paw Edema

In the dextran-induced paw edema model, male *Wistar* rats (*n* = 5 rats per group) were treated with vehicle, HEPB (200 mg/kg and 400 mg/kg), or cyproheptadine (10 mg/kg, by gavage). After 1 h of treatment, the animals received an intraplantar injection of 100 µL of dextran (1%; *w*/*v*) in the right hind paw and 0.9% saline in the contralateral paw. The paw volume was measured at 0, 30, 60, 90 and 120 min after injection of dextran with a plethysmometer. Edema volume was determined by the difference between right and left paw volume as previously described and adapted [12].

#### 3.1.5. Formalin Test

This test is an antinociceptive assay which represents a chemical nociception model that uses formalin solution. According to Hunskaar et al. [13], the mice (*n* = 6–8 animals per group) were treated with vehicle, HEPB (400 mg/kg), FR6-10 (250 mg/kg) or Morphine (10 mg/kg; subcutaneously). Briefly, the animal groups received the vehicle or the extract, 1 h or 30 min (for Morphine group) of the noxious stimuli [20 µL of a 1% formalin solution (0.92% formaldehyde) in saline] that was injected intraplantarly in the right hind paw. After that, the mice were individually placed under a glass cylinder (22 cm in diameter) and were observed from 0 to 5 min (neurogenic phase) and 15 to 30 min (inflammatory phase). The time that the animals spent licking the injected paws was measured as an indicator of nociception level.

#### 3.1.6. Acute Oral Toxicity

Acute oral toxicity was evaluated in female *Wistar* rats (*n* = 5 animals per group) according to Organization for Economic Co-operation and Development (OECD) Test Guideline 423 protocol. Each group was fasted for 12 h and then 2000 and 5000 mg/kg plant extracts (doses more likely to cause death) were administered. Immediately after HEPB administration, animals were placed individually in a cage with food and water ad libitum.

For behavioral changes evaluation, the animals were observed for a minute after extract administration at 0 min, 10 min,15 min, 30 min, 60 min, 2 h, 3 h, 24 h (1st day), 48 h (2nd day), and 72 h (3rd day). According to the test described by Malone et al. [11], the behavioral parameters that may be analyzed relating to stimulant activity were snout scratching, tremors, exophthalmia, attention, increased respiratory rate, paw licking, tail biting, arousal, spontaneous motor activity, lack of appetite, nasal discharge, piloerection, stereotyped movements, escape reaction, and convulsions. The parameters related to depressant activity were alienation of the environment, analgesia and anesthesia, ataxia, catatonia, decreased respiratory rate, decreased motility, decreased corneal reflex, apathy, dyspnea, response to touch, ptosis, sedation, and dorsal tone. Other parameters observed were aggressiveness, writhing, pupil diameter, diarrhea, cyanotic, hyperemic, pale ears, sweating, increased or decreased urination, grunting, tail tremors, tearing, coma, and death. As recommended by ethical statements, if symptoms related to prolonged seizures, coma or other severe conditions that may indicate heavy animal suffering were observed, animals may be euthanized.

After this period, the animals received food and water ad libitum and were observed and weighted for an additional 11 days to verify the possible occurrence of death [37].

For anti-edematogenic and antinociceptive tests, we defined the doses corresponding to 10% and/or 20% of the lowest non-toxic HEPB dose evaluated in the oral toxicity test. The FR6-10 dose was calculated according to the yielding percentage and the dose of HEPB 400 mg/kg.

#### 3.1.7. Statistical Analysis

Firstly, data normal distribution was verified by the Kolmogorov–Smirnov test. Besides, results were analyzed using one-way analysis of variance (ANOVA) for the formalin test. Repeated measures one-way ANOVA was performed for carrageenan-induced and dextran-induced paw edema tests. All statistical tests were followed by the Bonferroni post hoc test. *p*-Values less than 0.05 (*p* < 0.05) were considered to be indicative of significance. SigmaPlot 12.5 (San Jose, CA, USA) software was used for statistical analyses.

### 3.2. Collection, Identification and Preparation of Crude Extract

The leaves of *P. brasiliensis* were collected in Abaetetuba city (Pará state, Amazon region) in March of 2013. The region is located at latitude 01°43′25.7″ and longitude 048°52′50.3″ (geographic coordinates obtained using global positioning system [GPS] equipment). The botanical identification was performed by the specialist Dr. Silvane Tavares Rodrigues from Embrapa Amazônia Oriental (Pará-Brazil) and a voucher specimen was deposited in the IAN Herbarium of the Brazilian Agricultural Research Corporation of Eastern Amazon (Embrapa), under number 185501.

Dried and powdered leaves of *P. brasiliensis* (300 g) were extracted by maceration with ethanol [water:ethanol in the proportion of 30:70 (*v*/*v*); 70%] at room temperature for one week. The solution was filtered, and the solvent was removed under vacuum, yielding the hydroalcoholic extract of *P. brasiliensis* (HEPB, 62.0 g; which corresponds to 20.7% of the powder yield).

### 3.3. General Chemistry

Optical rotation was measured on an automatic polarimeter (Nova Instruments, model NO 1412, São Paulo, Brazil). The IR spectrum was obtained on a Shimadzu IR Prestige 21 FTIR spectrometer (KBr). Nuclear magnetic resonance (NMR) data were obtained on a Varian spectrometer (Mercury Plus 300, Palo Alto, CA, USA) and Bruker spectrometer (model DRX 400, Billerica, MA, USA). Chemical shift values were expressed in parts per million (δ) in CDCl_3_, CD_3_OD and C_5_D_5_N with tetramethylsilane (TMS) as an internal reference. Mass spectra were obtained on a Xevo G2S-Q-TOF 4K mass spectrometer equipped with an ESI source (Waters, Manchester, UK). The samples were examined by high performance liquid chromatography (HPLC Prominence, Shimadzu, Kyoto, Japan) composed of a binary solvent delivery and a photodiode-array (PDA) detector with reversed phase Gemini-C18 column 5 µm (4.6 mm × 250 mm, Phenomenex, Torrance, CA, USA). The compounds were isolated on a semi-preparative HPLC (LC 6, Shimadzu, Kyoto, Japan) equipped with dual channel UV detector and a reversed phase Gemini-C18 column 5 µm (10 mm × 250 mm, Phenomenex, Torrance, CA, USA) using a flow rate of 4.7 mL/min.

### 3.4. Isolation of Compounds

The HEPB (30 g) was chromatographed on a silica gel column eluted with increasingly polar mixtures: Hex:EtOAc (1:1), EtOAc, EtOAc:MeOH (8:2), EtOAc:MeOH (6:4), EtOAc:MeOH (4:6) and MeOH, resulting in 13 fractions (FR1-FR13). The fractions collected were analyzed on thin-layer chromatography (TLC) plates. Out of the 13 fractions, fractions FR7, FR8, FR9 and FR10 showed major spots representing the plant constituents and were in a large enough quantity for further purification. Fraction FR6 showed one spot and this yielded compound **7** (113.0 mg). The isolation of compounds was carried out using aliquots of 200.0 mg of each fraction FR7, FR8 and FR9, which were sonicated in 4.8 mL of methanol for 1 min. Next, 1.2 mL of H_2_O was added and sonicated again for 1 min. Solutions were subjected to solid phase extraction (SPE) in a C18 cartridge (Phenomenex, 1 g of stationary phase per 6 mL). After evaporation, the residues (about 160.0 mg of FR7, 142.0 mg of FR8 and 150.0 mg of FR9) were purified by semi-preparative HPLC, using an elution system consisting of H_2_O:ACN (73:27 *v*/*v*) yielding compounds **1** (44.0 mg) and **2** (9.0 mg) from FR9, compounds **2** (24.5 mg), **3** (7.5 mg) and **4** (7.0 mg) from FR8 and compounds **5** (30.0 mg) and **6** (34.0 mg) from FR7, respectively. In this sense, fractions from FR6 to FR10 (FR6-10) were pooled to perform the bioassays yielding 65% (*m*/*m*) of the HEPB.

#### 3.4.1. HPLC Profile

The sample (HEPB) at 1 mg/mL was prepared in H_2_O:MeOH (2:8 *v*/*v*) and the analysis was carried out on a reversed phase Gemini-C18 column 5 µm (4.6 mm × 250 mm). The binary solvent was composed of (A) water and (B) acetonitrile. The linear solvent gradient varied from 5 to 100% of B in 60 min at a flow rate of 1.0 mL/min. The injection volume was 20 µL and the compounds were detected at λ 200–400 nm.

#### 3.4.2. Compound **3**

5-*O*-β-d-glucopyranosyljusticidin B (**3**), a white amorphous solid; ${\left[\alpha \right]}_{D}^{25}$ + 150 (c 0.15, MeOH); IR (KBr): ν_max_ = 3370, 1774 and 1619 cm^−1^; ESI-TOF-MS (positive mode) found [M + H]^+^ at *m*/*z* 543.1503 (calculated for C_27_H_27_O_12_ at *m*/*z* 543.1503); ^1^H, ^13^C and 2D-NMR (CD_3_OD) spectroscopic data, see Table 1.

## Figures and Tables

**Table 1:** ^1^H (300 MHz) and ^13^C (75 MHz) NMR spectroscopic data (in CD_3_OD) of compound **3**.

Moiety	Position	3			
		δ_C_	δ_H_ (*J* in Hz)	HMBC (H to C)	COSY (H to H)
Aglycone moiety	1	140.4 ^a^			
	2	120.8			
	3	140.5 ^a^			
	4	116.9	8.45 s	2, 5, 10	12
	5	145.4			
	6	144.4			
	7	154.6			
	8	104.0	6.98 s	1, 7	OCH_3_-7
	9	129.8 ^b^			
	10	131.4 ^b^			
	11	172.3			
	12	70.1	5.43 s	3, 4, 11	4
	1′	130.3			
	2′	111.5	6.78 d (1.5)	3′, 4′, 6′	6′
	3′	149.0			
	4′	149.0			
	5′	109.0	6.95 d (7.8)	1′, 3′, 4′	
	6′	124.6	6.75 *dd* (1.5, 7.8)	2′	2′
	7′	102.6	6.03 d (1.2) 6.04 d (1.2)	3′, 4′	
	OCH_3_-6	62.0	3.97 s	6	
	OCH_3_-7	56.1	3.74 s	7	8
Sugar moiety	1″	105.7	5.16 d (7.8)	5	
	2″	75.7	3.65 m		
	3″	77.9	3.49 m		
	4″	71.3	3.46 m		
	5″	78.3	3.20 m		
	6″	62.2	3.69 m		

^a^ and ^b^ assignments may be interchanged within each column.

## Supplementary Materials

Supplementary materials are available online.
