# Supplementary material for: Anti-Inflammatory and Antinociceptive Studies of Hydroalcoholic Extract from the Leaves of Phyllanthus brasiliensis (Aubl.) Poir. and Isolation of 5-O-β-d-Glucopyranosyljusticidin B and Six Other Lignans
Source: Molecules. 2018 Apr 18;23(4):941. doi: 10.3390/molecules23040941 (PMC6017254; doi:10.3390/molecules23040941)
Supplement: Supplementary file 1 [file molecules-23-00941-s001.pdf]

Anti-inflammatory and antinociceptive studies of hydroethanolic extract from the leaves of *Phyllanthus brasiliensis* (Aubl.) Poir. and isolation of 5-O-β-D-glucopyranosyljusticidin B and other six lignans

Luziane Borges [1], Raimundo Negrão-Neto [1], Sônia Pamplona [1], Luanna Fernandes [2], Mayra Barros [2], Enéas Fontes-Júnior [2,3], Cristiane Maia [2,3], Consuelo Y. e Silva [3,*] and Milton N. da Silva [1]

[1] Programa de Pós-Graduação em Química, Instituto de Ciências Exatas e Naturais, Universidade Federal do Pará, Rua Augusto Corrêa, 01, 66075-110, Belém, Pará, Brazil; negraoneto@yahoo.com.br; luziane_borges22@yahoo.com; sgpamplona@yahoo.com.br; yumilton@yahoo.com.br

[2] Programa de Pós-Graduação em Ciências Farmacêuticas, Instituto de Ciências da Saúde, Universidade Federal do Pará, Rua Augusto Corrêa, 01, 66075-110, Belém, Pará, Brazil; luannafe@hotmail.com; mayraarouckbarros@gmail.com; efontes@ufpa.br, crismaia@ufpa.br

[3] Faculdade de Farmácia, Instituto de Ciências da Saúde, Universidade Federal do Pará, Rua Augusto Corrêa, 01, 66075-110, Belém, Pará, Brazil; yumikoyoshioka@yahoo.com.br

*Correspondence: yumikoyoshioka@yahoo.com.br; Tel.: +55-091-3201-7365

Contents

Fig.S1 Infrared spectrum of compound 3.. 2
Fig.S2 MS spectrum of compound 3.. 2
Fig.S3 1H-NMR of compound 3.. 3
Fig.S4 13C-NMR of compound 3... 3
Fig.S5 1H-1H COSY of compound 3…….. 4
Fig.S6 HETCOR of compound 3……………………………………………….. 5
Fig. S7 HMBC of compound 3……... 6

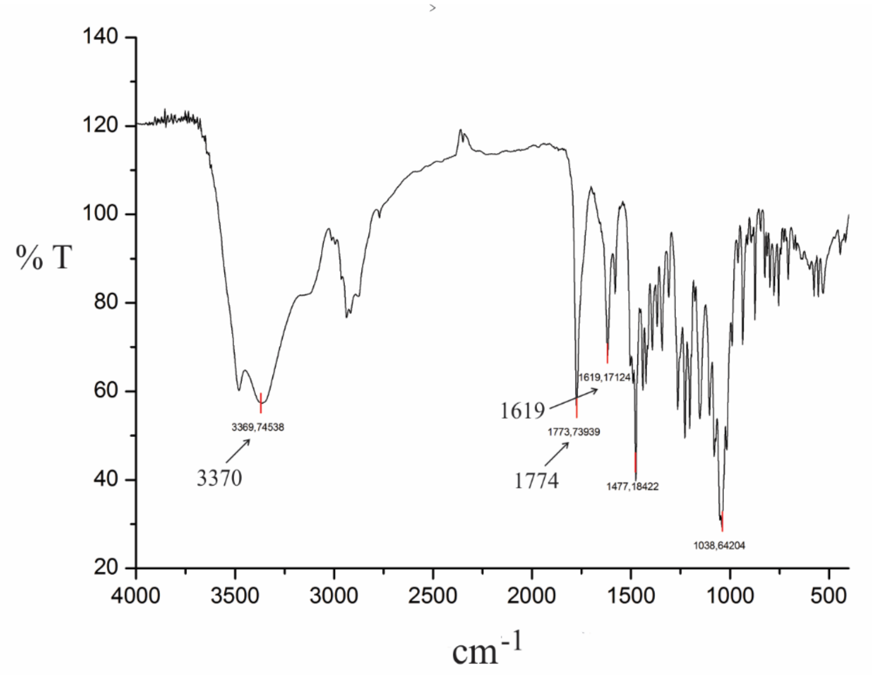

Fig.S1 Infrared spectrum of compound 3.

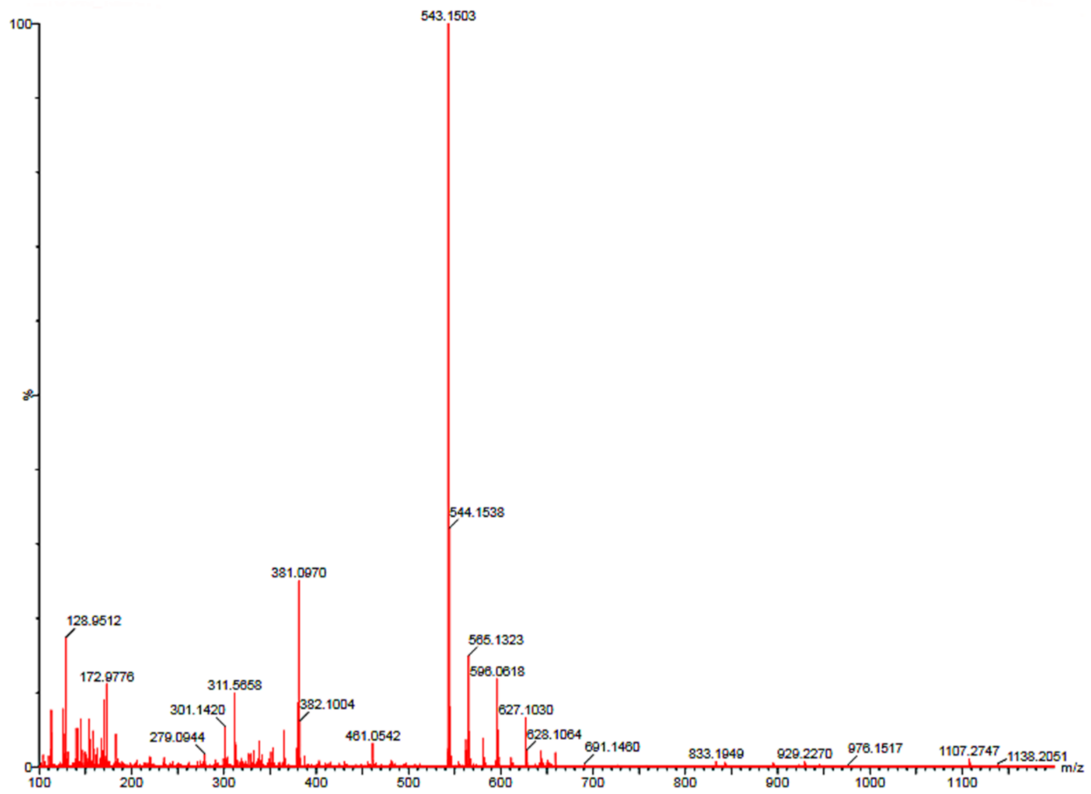

Fig.S2 MS spectrum of compound 3.

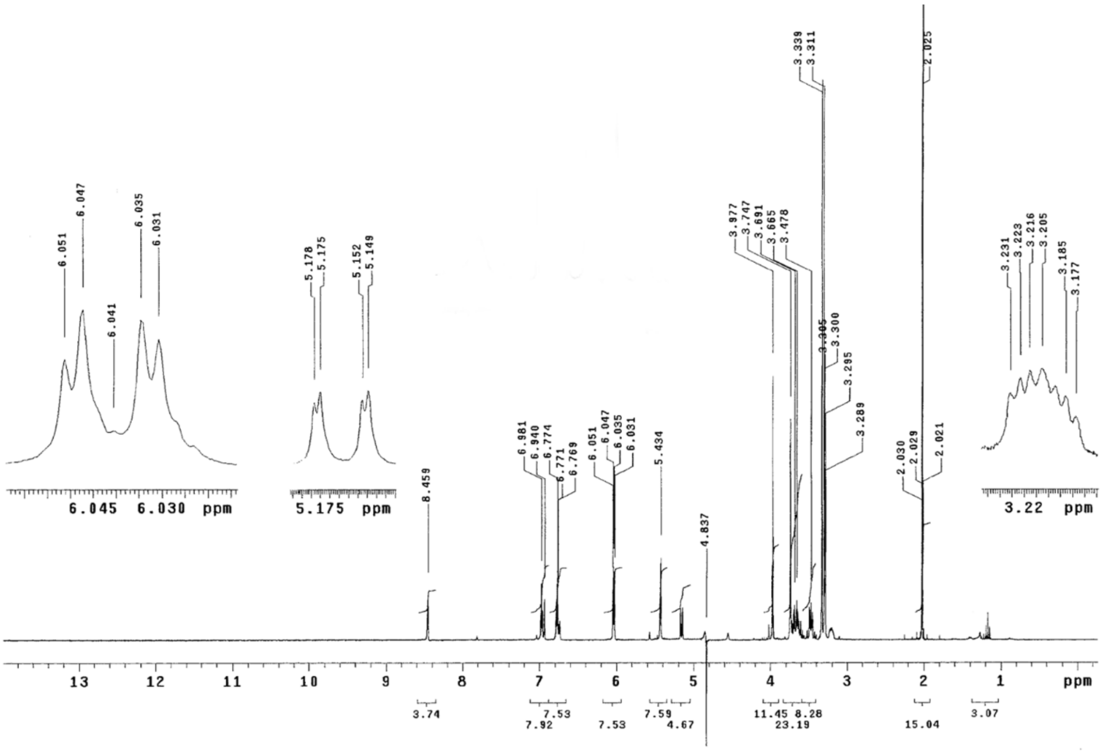

Fig.S3 1H-NMR of compound 3.

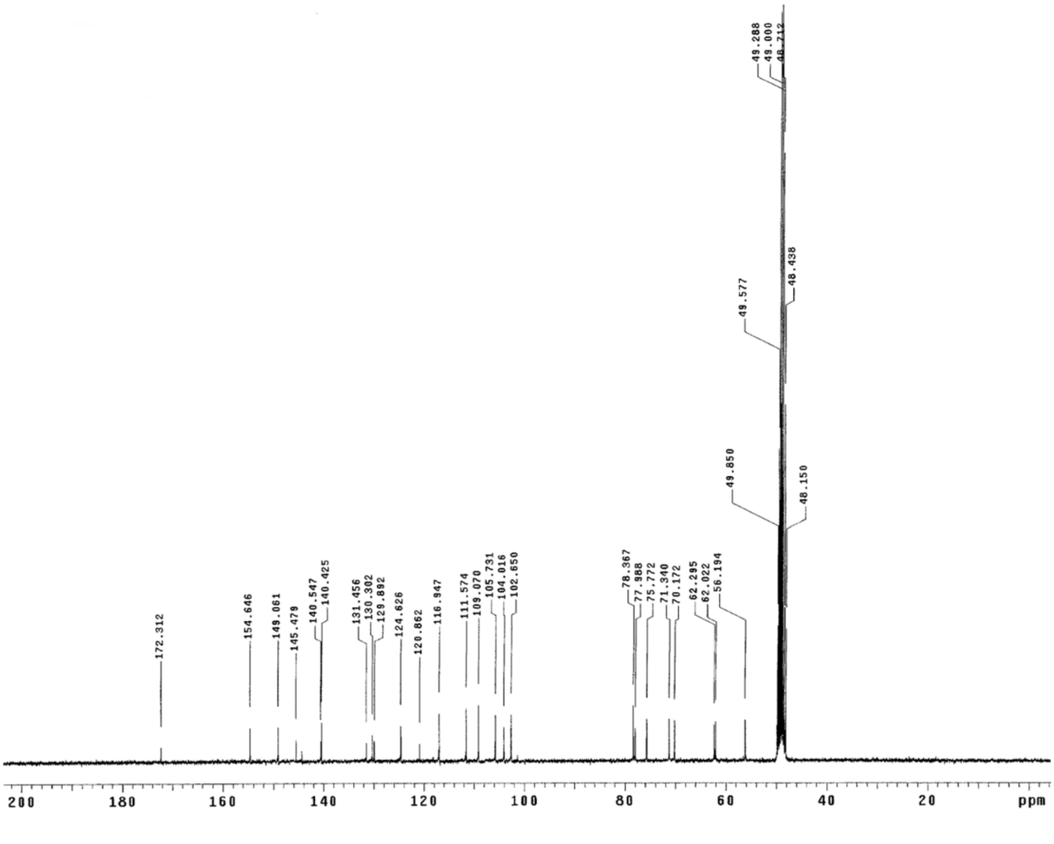

Fig.S4 13C-NMR of compound 3.

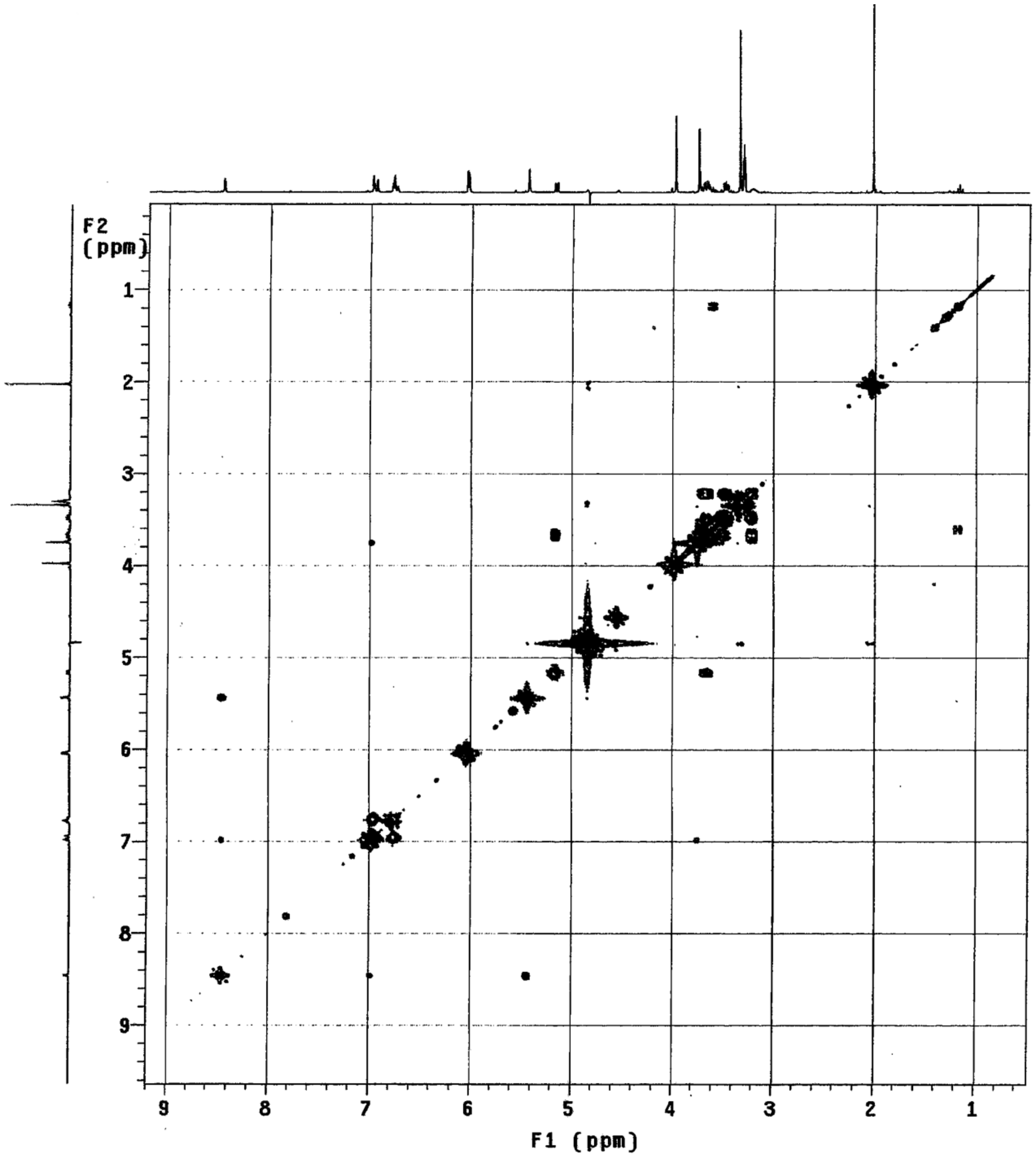

Fig.S5 1H-1H COSY of compound 3.

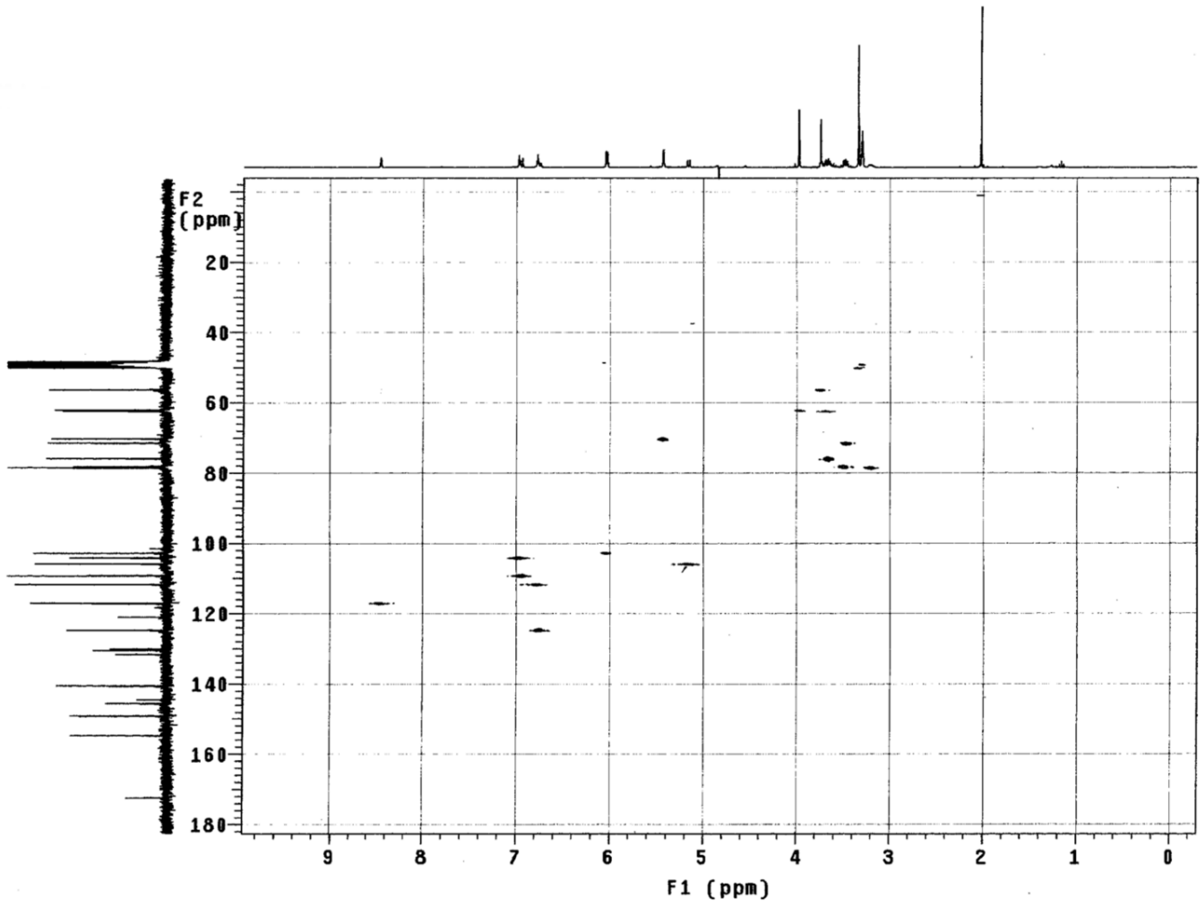

Fig.S6 HETCOR of compound 3.

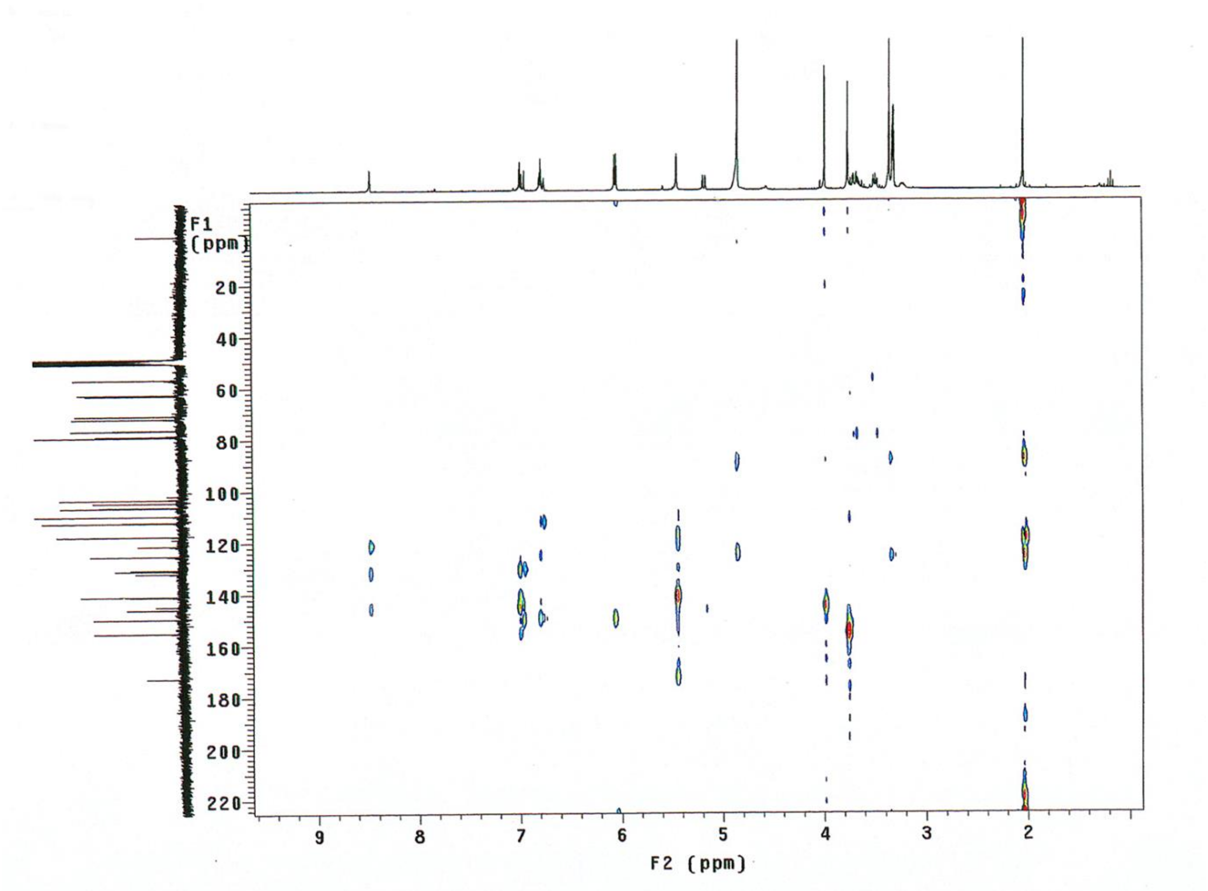

Fig. S7 HMBC of compound 3.
